# Preclinical characterization of CPL302-253, a selective inhibitor of PI3Kδ, as the candidate for the inhalatory treatment and prevention of Asthma

**DOI:** 10.1371/journal.pone.0236159

**Published:** 2020-07-23

**Authors:** Paweł Gunerka, Kamila Gala, Martyna Banach, Jakub Dominowski, Joanna Hucz-Kalitowska, Krzysztof Mulewski, Agnes Hajnal, Endre G. Mikus, Damian Smuga, Marcin Zagozda, Krzysztof Dubiel, Jerzy Pieczykolan, Beata M. Zygmunt, Maciej Wieczorek

**Affiliations:** 1 CelonPharma Innovative Drugs Research & Development Department, Celon Pharma S.A., Lomianki, Poland; 2 LabMagister Training and Science Ltd., Budapest, Hungary; Wayne State University, UNITED STATES

## Abstract

Asthma is a common chronic inflammatory disease. Although effective asthma therapies are available, part of asthmatic population do not respond to these treatment options. In this work we present the result of development of CPL302-253 molecule, a selective PI3Kδ inhibitor. This molecule is intended to be a preclinical candidate for dry powder inhalation in asthma treatment. Studies we performed showed that this molecule is safe and effective PI3Kδ inhibitor that can impact many immune functions. We developed a short, 15-day HDM induced asthma mouse model, in which we showed that CPL302-253 is able to block inflammatory processes leading to asthma development *in vivo*.

## Introduction

Asthma is a common chronic inflammatory disorder with a spectrum of recurrent and reversible respiratory symptoms like cough, wheezing and shortening of breath. Over the course of this disease, multiple immune cell types act together with epithelial cells in response to the stimulus, leading to chronic inflammation, airway hyperresponsiveness, mucus overproduction, remodelling and narrowing of the airway. One of the risk factors determining susceptibility to asthma is the exposure to inhaled allergens, the small particles that reach the respiratory track with the air and trigger inflammatory cascade leading to the disease progression [1, 2].

The classical view of asthma development postulates that during the first encounter with an allergen the organism is sensitized [2]. Antigen presenting cells capture antigens from lung epithelial surface, process it and present in the draining lymph node to naïve CD4 T cells leading to effector and memory T helper (Th) cells generation. During second exposure to allergen, strong immune response regulated by cytokine-producing Th cells is induced, in which different innate immune cells take part, what causes disease symptoms. There are different subsets of Th cells. The role of Th2 cells in asthma is best described. Th2 cells produce IL-4, IL-5 and IL-13, cytokines responsible for airway hyperresponsiveness, goblet cells metaplasia and mucus production. Th2 cells also orchestrate eosinophil maturation, their migration to the lungs and degranulation. Release of granules leads to activation of neutrophils and basophils as well as epithelial surface damage. All these events lead to induction of asthma symptoms. Recently, other inflammatory mechanisms leading to asthma development have been identified [2, 3]. In the innate cell-mediated asthma, inflammation is induced during the first encounter with an allergen, which activates Pathogen Associated Molecular Patterns (PAMP) receptors present on epithelial cells and causes epithelial layer mechanical disruption. This induces production of IL-25, IL-33 and thymic stromal lymphopoietin by epithelial cells and activation of Innate Lymphoid Cells (ILC), which in turn initiates inflammatory cascade involving multiple immune cell types. Numerous studies in human cohorts showed that involvement of immune cell types and effector molecules, as well as mechanism leading to disease development, differ significantly among patients. There are also considerable differences in response to available treatment options in the asthmatic population, which includes patients suffering from steroid resistant asthma. This lead to the postulate that asthma cannot be considered as a single disease but rather clinical syndrome, characterized by a set of symptoms caused by multiple pathological mechanisms.

Phosphoinositide 3-kinase (PI3K)δ, the member of the class I PI3K family, is an important signalling molecule which regulates proliferation, differentiation, migration and survival of immune cells [4]. It is expressed, in contrast to other isotypes of PI3K, almost exclusively in immune cells, which makes it an attractive target for inflammatory disease treatment. PI3Kδ regulates multiple processes involved in allergic asthma development. It is responsible for activation, differentiation and cytokine expression by Th2 cells, as well as ILC. Moreover, PI3Kδ regulates activation and allergy relevant antibody (e.g. IgE) production by B cell and is important for basophil activation. Finally, PI3Kδ regulates eosinophil migration and accumulation in the lungs. Recent reports pinpoint that allergic asthma is regulated by PI3Kδ expressed by lung epithelial cells [5]. All these features makes PI3Kδ an attractive target for asthma treatment in patients suffering from asthma resistant to currently available therapies. Up to date several PI3K isoform-specific inhibitors were developed and are in the market toward blood cancers. The first in class inhibitors registered for hematologic cancer therapy are idelalisib (specific for PI3Kδ), duvelisib (specific for PI3Kδ and γ) and copanlisib (specific for PI3Kα and δ) [6–8]. However, the data indicating toxicity of the above-mentioned drugs exclude their usage in patients not suffering from a life-threating disease. Therefore, development of PI3Kδ inhibitors as a therapy for inflammatory disease should concentrate on reduction of compounds toxicity. The adverse effect is often caused by drug low specificity or systemic action and can be limited by increasing drug specificity or restricting drugs site of action [9]. Respiratory drug delivery, like inhalation therapy is one of the possible mechanisms used to decrease toxicity and side effects of lung disease treatment. This inhalation approach is successfully used for decades in treatment of asthma limiting the systemic side effects of corticosteroids [10]. Currently there are several PI3Kδ inhibitors under development in immune-related disorders, all for systemic administration [11–13]. Nemiralisib was the only molecule tested in clinical trials which was developed for inhalation administration in lung disease, however recently development of this molecule has been stopped [14].

House Dust Mite (HDM) is a member of Pyroglyphidae family found in association with dust in human houses. It is a common allergen and it is estimated that, depending on geographic region, 50 to 85% people suffering from asthma, are allergic to HDM [15]. Similar to ovalbumin or chicken egg albumin (OVA), the common allergen used to study immune response in allergy and asthma animal models, HDM sensitization leads to Th2 cell induction and eosinophil infiltration to the lungs. In contrast to OVA, HDM is a complex antigen that triggers both adaptive and innate arms of immune system. HDM poses protease activity which is necessary to induce mechanical disruption of epithelial cells. It is also an agonists of PAMPs which directly induce endothelial IL-33 and other cytokine production. Therefore HDM can lead to group-2 ICL mediated asthma induction. It is believed that, due to numerous similarities between rodent and human immune response to HDM exposure, HDM animal models will improve translatability in asthma drug discovery. There are several HDM sensitization protocols differing in route of administration, exposure as well as study duration [16, 17]. While the first encounter with the antigen may occur through parenteral or respiratory route, in further steps of the study HDM is administrated multiple times over the time period of a few to a dozen or so weeks. Development of shorter HDM sensitization protocol would facilitate the preclinical development of asthma drugs.

In current work, we present the result of development of CPL302-253 molecule which is a selective PI3Kδ inhibitor, intended to be a preclinical candidate for dry powder inhalation in asthma treatment and prevention. The *in vitro* tests we performed showed no off-target effects which could lead to toxicity *in vivo*. The *in vitro* studies on primary cells showed that CPL302-253 is an effective inhibitor that can impact many immune functions. The studies in 15-day HDM induced asthma mouse model showed that CPL302-253 is able to block inflammatory processes leading to asthma development *in vivo*.

## Materials and methods

### Compound preparation

For the in vitro assays the stock CPL302-253 solution was prepared from solid compound dissolved in DMSO at 10 mM. Serial dilutions were performed in the working buffers or culture media depending on experiment. In all *in vitro* experiments DMSO vehicle controls were included.

For the in vivo experiments the CPL302-253 was dissolved in sterile and pyrogene free 100mM phosphate buffer (pH 7.4) with 0.2% Tween 80.

### Kinase assays IC50 determination

The potency and selectivity of CPL302-253 against different isotypes of class I PI3K were assessed using ADP-Glo Kinase Assay (Promega) according to manufacturer’s protocol. PI3Kα, PI3Kβ, PI3Kδ, PI3Kγ were purchased from Merck Millipore. The inhibition of examined kinases activity was evaluated by measuring their ability to converse ATP to ADP in the presence of decreasing doses of tested compound (from 400 μM to 0,00004 μM). Kinases were mixed with 1 mM PIP2:PS substrate (Phosphoinositol-4,5-bisphosphate (PIP2) lipid vesicles with phosphoserine (PS), Thermofisher Scientific) and 30 μM ATP in an assay buffer (50 mM HEPES pH 7.5,50 mM NaCl, 3 mM MgCl2, 25 μg/ml BSA). The mixture was incubated in the dark for 1 h at 25°C (PI3Kα and PI3Kδ) or at 30°C (PI3Kβ, PI3Kγ). Next ADP-Glo reagent 1 was added to stop the reaction and remove any remaining ATP. The samples were incubated for 40 min at 25°C. Subsequently the ADP-Glo reagent 2 was added and reactions were incubated for 40 min at 25°C. Finally, luminescence was assessed and the IC50 was calculated.

### Assessment of compound interaction with kinases

The inhibitor-kinase binding affinity was assessed in KINOMEscan™ assay (Discoverex) according to service provider’s protocol. In brief, in this binding assay the studied compound in solution is incubated with DNA-tagged kinase and kinase active-site directed ligand bound to the beads. The compound competes for binding to kinase with the immobilized ligand. After incubation beads are removed from the solution and kinase bound to ligand is quantified by qPCR. The strength of compound binding is determined based on its ability to block kinase-ligand binding [18]. 11-point 3-fold serial dilution of CPL302-253 was used in order to determine its binding constants (Kd) against different isotypes of class I PI3K. The single concentration of CPL302-253 was used in order to determine its specificity in abroad panel of 468 human kinases. The measure of compounds affinity to each kinase in the panel was the percentage of kinase activity inhibition by tested ligand in comparison to the control.

### BioMAP Profiling

BioMap Profiling study was performed by DiscovereX according to service provider’s protocol. In brief cultures or co-cultures of human primary cells were treated with sets of stimulators specific for cell subsets. Cell types and stimulators in each system were as follows: 3C system (HUVEC stimulated with IL-1β, TNFα and IFNγ), 4H system (HUVEC stimulated with IL-4 and histamine), LPS system (PBMC and HUVEC stimulated with LPS), SAg system (PBMC and HUVEC stimulated with TCR ligands), BT system (CD19 B cells and PBMC stimulated with αIgM and TCR ligands), BF4T system (bronchial epithelial cells and HDFn stimulated with TNFα and IL-4), BE3C system (bronchial epithelial cells stimulated with IL-1β, TNFα and IFNγ), CASM3C system (coronary artery smooth muscle cells stimulated with IL-1β, TNFα and IFNγ), HDF3CGF system (HDFn stimulated with IL-1, TNFα, IFNγ, EGF, bFGF and PDGF-BB), KF3CT system (keratinocytes and HDFn stimulated with IL-1β, TNFα, IFNγ and TGFβ), MyoF system (differentiated lung myofibroblasts stimulated with TNFα and TGFβ) and lymphsystem (HUVEC and M1 macrophages stimulated with Zymosan). The assays included positive and negative controls as well as vehicle control. The expression of biomarkers specific for each system was measured by ELISA and other techniques. The results are shown as log-transformed ratio of the biomarker readouts for the drug-treated sample to vehicle controls. The grey area parallel to the x-axis indicates the 95% significance region calculated based on historical vehicle controls. Biomarker activities are considered as changed when 2 or more consecutive concentrations change in the same direction relative to vehicle controls, are outside of the significance envelope, and have at least one concentration with an effect size > 20%. The antiproliferative effect is indicated by a thick grey arrow [19].

### SafetyScan44

SafetyScan44 assay was performed by DiscoverX according to service provider’s protocol.

### Normal Human Bronchial Epithelial (NHBE) cells cytotoxicity studies

The study was performed by Cyprotex according to service provider’s protocol. In brief NHBE cells were plated on 96-well plate. The cells were dosed with test compound at 0.0457, 0.137, 0.412, 1.24, 3.7, 11.1, 33.3, 100 μM and incubated for 48 h. The cells were loaded with dye or antibody relevant for each health marker and scanned using fluorescent cell imager. The following parameters were studied: cell count, nuclear size, DNA structure, mitochondrial mass, mitochondrial membrane potential, oxidative stress, glutathione content. The baseline for each parameter was determined based on vehicle control sample. The software calculates the percentage of cells that are low or high responders (depending on the biological significance of a particular readout). The vehicle control wells are then used to determine significance limits for wells that have a greater than expected fraction of low or high responders. The minimum effective concentration was determined from the lowest concentration whose mean value exceeds the significance level and provided either a clear dose-response relationship or at least two consecutive concentration points past the significance level.

### PBMC isolation

Human PBMC were isolated from buffy coats of healthy donors by density gradient centrifugation in Ficoll-Paque Plus (GE Healthcare). After isolation, cells were frozen in FBS (Biowest) with 10% DMSO (Sigma-Aldrich). The buffy coats were obtained from Regional Blood Donation and Blood Medicine Center in Warsaw.

### B lymphocyte activation assay

B cells were isolated from human frozen PBMCs using EasySep Human CD19 Positive Isolation Kit (StemCell Technologies) according to manufacturers’ protocol. The cells were seeded in AIM V medium (Gibco). The cells were pre-treated for 1 h with different concentrations of CPL302-253 and subsequently stimulated 1μg/ml ODN2006 (Invivogen) and 10μg/ml αIgM F(ab’)_2_(Jackson ImmunoResearch). After 72 hours of culture the cells were stained with antibody againstCD25 (BC96, eBioscience) and ki-67 (Clone 20Raj1, Invitrogen) and analysed on FACSCalibur.

### CD8 T cell activation

CD8 T cells were isolated from human frozen PBMC using EasySep Human CD8 T cell Isolation Kit (StemCell Technologies) according to manufacturer’s protocol and cultured in AIM V medium (Gibco). To cell culture different concentrations of CPL302-253 were added. The cells were activated with Human T Cell Activation/Expansion Kit (Miltenyibiotec) according to manufacturer’s protocol. IFNγ production was measured after 48 h of activation in culture supernatants using IFNγ Human Uncoated ELISA Kit (Invitrogen) according to manufacturer’s protocol.

### A549 CD8 T cell activation

A549 cells were seeded into 48-well plate. CD8 T cells were isolated from frozen human PBMC using EasySep Human CD8 T cell Isolation Kit (StemCell Technologies) according to manufacturer’s protocol. A549 were co-cultured with CD8 T cells at 1 to 5 ratios for 72 h. In some cultures, CD8 cells were activated with Human T Cell Activation/Expansion Kit (Miltenyibiotec) according to manufacturer’s protocol. After 72h CD8 cells were removed by rinsing with PBS. Adherent A549 cells were suspended in 100 μl RIPA and IL-33 was assessed in cell lysates using IL-33 Human Uncoated ELISA Kit (Invitrogen). Due to high variability in cell activation the data were normalized (measure for each experimental condition was divided by average of measures for all experimental conditions in the experiment).

### Neutrophils IL-8 production

Neutrophils were isolated using EasySep Direct Human Neutrophil Isolation Kit (StemCell Technologies) from fresh whole blood of healthy donors by magnetic bead isolation according to manufacturer’s protocol. The cells were cultured in AIM V medium (Gibco). After 30 min pre-treatment with CPL302-253 (1 μM) neutrophils were stimulated with Cigarette Smoke Extract (CSE) (Murty Laboratories) at 100 μg/ml or LPS from E. coli O55:B5 (Sigma-Aldrich) at 1 μg/ml. After 6 h of incubation the culture supernatants were collected and IL-8 production was measured using IL-8 Human Uncoated ELISA Kit (Invitrogen) according to manufacturer’s protocol

### Neutrophil ROS production

Neutrophils were isolated using EasySep Direct Human Neutrophil Isolation Kit (StemCell Technologies) from fresh whole blood of healthy donors according to manufacturer’s protocol. Cells were cultured in AIM V medium (Gibco). Cells were loaded for 30 min at 37°C in humidified incubator in the presence of 5% CO_2_ with DCFDA (2’,7’–dichlorofluorescein diacetate), a fluorescent ROS indicator. Subsequently neutrophils were treated for 15 min with CPL302-253 and dexamethasone. In the next step 20 ng/ml of human GM-CSF (StemCell Technologies) was added to prime cells. 15 min later 1 μg/ml LPS from E. coli O55:B5 (Sigma-Aldrich) was added to induce ROS generation. The ROS accumulation in the neutrophils was assessed on FACSCalibur 2 h after LPS stimulation.

### Neutrophil viability assessment

Neutrophils were isolated using EasySep Direct Human Neutrophil Isolation Kit (StemCell Technologies) from fresh whole blood of healthy donors according to manufacturer protocol (StemCell Technologies). The cells were cultured in AIM V medium (Gibco). After 30 min pre-treatment with test compound neutrophils were stimulated with 100 ng/ml LPS from E. coli O55:B5 (Sigma-Aldrich). After 24 hours of incubation cells were stained with annexin V (Thermofisher) and Propidium Iodide (PI) (Thermofisher) according to manufacturer’s protocol. Cell viability was assessed on FACSCalibur.

### Basophils activation

The study was performed using Flow CAST Basophil Activation Test (Buhlmann) according to manufacturer’s protocol with modifications. In brief to U bottom 96-well plate 50 μl of stimulus solution (stimulating antibody specific for the high-affinity IgE receptor FcεRI) or assay bufferwas added. Subsequently to some wells, 50 μl of inhibitor at different concentrations in assay buffer was added. In the next step 50 μl of EDTA collected fresh human blood was added to each well followed by 50 μl of staining reagent (diluted 2:3 in assay buffer). The cells were incubated for 25 min at 37°Cin humidified incubator in the presence of 5% CO_2_. After incubation cells were centrifuged and erythrocytes were lysed with pre-warmed (18–28°C) Lysing Reagent. After centrifugation cells were suspended in Wash Buffer and analysed by FACSCalibur. Data were analysed using CellQuestPro and FlowJo software.

### Metabolic stability

Assessment of metabolic phase I stability in mouse (CD-1) and human microsomes (ThermoFisher Scientific) was performed on 96-well non-binding plates (Greiner) at 1 μM concentration for both verapamil (positive control) and CPL302-253. Unless otherwise stated, all chemicals and materials were ordered from Merck Life Science. Each biological replicate was prepared in triplicates. Briefly, compounds were incubated in 100 mM potassium phosphate buffer with microsomes (0.5 mg/mL) and NADPH (1–1.2 mM) on a plate shaker (500 rpm) in dark at 37 ⁰C. 4x solution of NADPH, cofactor for metabolic enzymes, was prepared directly prior to the experiment by reducing NADP with G6P dehydrogenase (13.2 mM MgCl2, 13.2 mM G6P, 5.2 mM NADP, 3.2 U/mL G6P dehydrogenase, 20 min at 30 ⁰C, 500 rpm). The negative control contained buffer instead of NADPH solution. Samples were collected at 0, 10, 20 and 40 min or 0 and 40 min for the negative and double negative controls. The reaction was stopped by protein precipitation in 2 volumes of ice cold MeOH with 200 nM imipramine (as an internal standard for LC-MS analysis). Then, the extract was mixed (1 min, 1000 rpm), filtered through 0.22 μm filter on 96-well plate vacuum manifold and subjected to LC-MS analysis. Metabolic stability in mouse and human cryopreserved hepatocytes was performed in suspension on 96-well tissue culture plates at 1 μM concentration in Williams medium E with CM4000 supplements (ThermoFisher Scientific). Cells were thawed according to the manufacturers manual, counted and preincubated for 30 min in a CO2 incubator (5% CO2, 37 ⁰C, 5 · 104 cells/well). For the negative control cells were thermally inactivated for 5 min at 95 ⁰C. Similarly to microsomal stability, experiments were done in technical triplicates, with the additional time point at 80’. Samples for LC-MS analysis were prepared as described for microsomes. Intrinsic clearance (*Cl*_int_) was calculated using the rate of compound disappearance from the reaction. The elimination rate constant (*k*) was calculated as a module of slope of the linear regression from ln [substrate] vs time. Then, clearance was calculated as *Cl*_int_ = *k*/*d*, where *d* is protein or cell density for microsomal and hepatocyte stability, respectively.

### LC-MS method for metabolic stability

LC-MS analysis for metabolic stability assessment was performed using a UHPLC 1290 Infinity II (Agilent) with Ultivo Triple Quad MS (Agilent) or Triple Quad 6460 (Agilent) in the positive mode and multiple reaction monitoring (MRM) mode. The other MS parameters were as follows: gas temperature 350°C, gas flow rate 11 L/min, nebulizer 45 psi, sheath gas temperature 375 ⁰C, sheath gas flow rate 11 L/min, capillary voltage 3500 V, nozzle voltage 500 V. MRM transitions for CPL302-253 were 475.3 → 419 (CE 21 V) and 475.3 → 155 (CE 21 V) and 475.3 → 99 (CE 33 V) at fragmentor 170 voltage. MRM transitions for imipramine were 281.2 → 86.1 (CE 13 V) and 281.2 → 58.1 (CE 45 V) at fragmentor voltage 75 V. MRM transitions for verapamil 455.3 → 165.0 (CE 37 V) and 455.3 → 150.0 (CE 45 V) at fragmentor voltage 158 V. LC separation was achieved using an ACQUITY UPLC BEH C18 50 x 2.1 mm, 1.7 μm column (Waters) at 50°C. The mobile phases were (A) 10 mM ammonium acetate pH 5.4 in water and (B) methanol. Samples were eluted using a timed, linear gradient from 30% B at 0 min to 90% B at 1.5 min then a hold 90% B to 2.5 min, after it back to initial 30% B to 2.6 min. The columns was equilibrated for up to 5 min. The flow rate was 0.5 mL/min. The injection volume was 3 μL.

### Pharmacokinetic (PK)

Female Balb/c mouse (20–25 g bodyweight) were purchased from Nofer Institute (Lodz, Poland). Animals were given 0.3 mg/kg or 1 mg/kg single dose of CPL302-253 by intratracheal instillation. The compound was suspended in 0.2% DMSO in saline. 5 animals per time-point were sacrificed 10 min, 30 min, 1 h, 2 h, 4 h, 7 h and 12 h after compound administration. The blood was collected by heart puncture to K2EDTA containing tubes and plasma samples were prepared by centrifugation. The Bronchoalveolar Lavage Fluid (BALF) was collected by flushing the lungs 2 times with 1 ml of saline. The lungs were homogenized in 10% DMSO solution in water. 3ml of the solution were used for 1 g of lung tissue. The samples were purified using acetonitrile and concentration of CPL302-253 was measured by LC-MS/MS with internal standard. The concentration of the compound is given per ml of plasma and BALF or g of lung tissue.

### LC-MS method for PK

LC-MS analysis of PK samples was performed using 1290 Infinity II UPLC (Agilent) with 6460 Triple Quad MS (Agilent) in the positive mode and MRM mode. The other MS parameters were as follows: gas temperature 320°C, gas flow rate 11 L/min, nebulizer 55 psi, curtain gas temperature 330°C, curtain gas flow rate 11 L/min, capillary voltage 5500 V, nozzle voltage 500 V. MRM transitions for CPL302-253 were 475.0 → 419.0 (CE 17 V), 475.0 → 333.0 (CE 30 V) and 475.0 → 155.0 (CE 17 V) at fragmentor voltage 140 V. MRM transitions for donepezil were 380.5 → 287.9, 380.5 → 243.5 at fragmentor voltage 156 V and CE 24V. LC separation was achieved using an ACQUITY UPLC BEH C18 50 x 2.1 mm, 1,7 μm column (Waters) at 50°C. The mobile phases were (A) 10 mM ammonium acetate pH 8.2 in water and (B) acetonitrile:methanol (1:2, v/v). Samples were eluted using a timed, linear gradient from 10% B at 0.3 min to 95% B at 1.5 min, then back to 10% at 2.5 min. The column was equilibrated for up to 5 min. The flow rate was 0.4 mL/min. The injection volume was 5 μL. Calibration curve was prepared in the range from 1 ng/mL to 2000 ng/mL by spiking 45 μL of plasma with 5 μL of concentrated standard solution. All plasma based standard solutions and studied samples (50 μL) were subsequently extracted with 150 μL of acetonitrile containing donepezil as internal standard and centrifuged to provide supernatant ready for analysis.

### HDM-steroid insensitive asthma mice model

Balb/c female mice (18–25 g bodyweight) were purchased from Charles River Ltd. The Complete Freund’s Adjuvant (CFA)(Sigma-Aldrich) was mixed with House Dust Mite (HDM) lyophilized extract (Stallergenes Greer Ltd.) in a ratio of 100 μg HDM to 150 μl CFA. The mixture was vortex intensively and sonicated for 30 min in an ultrasonic bath at room temperature. On Day 0 animals were injected with the CFA/HDM mixture by s.c. route. Control animals received phosphate buffered saline (PBS). On Day 12, 13 and 14 the mice were anaesthetized by intramuscular ketamin/xilazine mixture and treated by intranasal (i.n.) route (2mL/kg) with CPL302-253 or saline. On Day 14, 60 min after CPL302-253 administration the animals were challenged by i.n. route with HDM in sterile saline (2 ml/kg; 100 μg/ml) or saline. On Day 15 the animals were sacrificed by the overdose of pentobarbital and BAL was collected. BAL samples were centrifuged and cell smears were prepared from the cell pellet by Cytospin cytocentrifuge (200 rpm for 4 min), fixed with Microscopy Hemacolor fixing solution (Merk KGaA, Germany) and stained with Microscopy Hemacolor Stain I-II (Merk KGaA, Germany). Eosinophils were counted using standard morphologic criteria. The differential counts on at least 100 cells per sample were done. IL-33 levels were measured in BALF using Mouse/Rat IL-33 Quantikine ELISA Kit (R&D Systems) from the supernatant of the BAL fluid. The protocol has been approved by the Hungarian Food Chain Safety and Animal Health Directorate (PE/EA/1306-7/2017) and was carried out in accordance with European Directive 86/609/EEC.

### Statistical data analysis

Statistical analysis was performed using GraphPad Prism software (version 7). Statistical significance between the mean of treated groups was compared to the mean of positive control group with one-way ANOVA with Dunnett correction. p-value <0.05 was regarded as significant response. * p< 0.05; ** p<0.01; *** p<0.001; **** p<0.0001. The statistical significance between positive and negative controls was determined using unpaired T-test. p-value <0.05 was regarded as significant response. # p< 0.05; ## p<0.01; ### p<0.001; #### p<0.0001.

## Results

**Fig 1:**
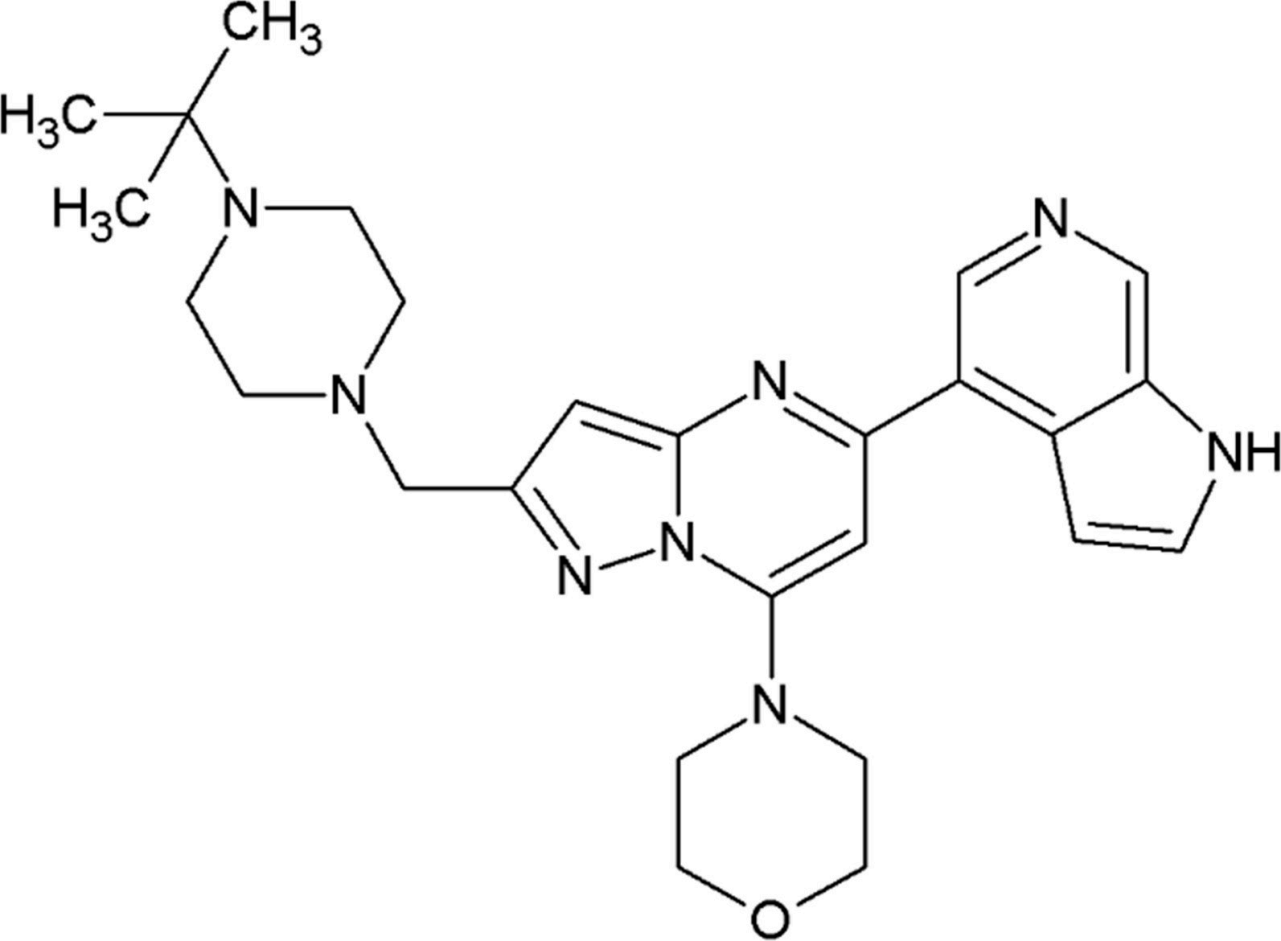
CPL302-253 chemical structure.

CPL302-253 is a novel derivative of 7-(morpholin-4-yl) pyrazolo [1,5-a] pyrimidine substituted with 1H-pyrrolo [2,3-c] pyridine and tert-butylpiperazine (Fig 1).

The selectivity and potency of CPL302-253 were measured against all class I PI3K isoforms (Table 1). The results obtained from KINOMEScan showed that the compound is highly potent both in blocking kinase activity in biochemical assays as well as in binding to PI3Kδ. It exerts its effect at concentrations as low as few nM. CPL302-253 is also very selective inhibitor of PI3Kδ isoform, as it influences other isoforms of class I PI3K at concentrations at least two orders of magnitude higher.

**Table 1:** CPL302-253 selectivity and potency in kinase assays. The CLP302-253 IC50 for the enzymatic activity of recombinant α, β, γ and δ PI3K isoforms was determined in ADP-Glo Kinase assay. The assay was performed at least 3 times. The CPL302-253 binding affinity was assessed in KINOMEScan competitive binding assay. Kd were calculated with a standard dose-response curve using the Hill equation.

PI3K isoform	δ	Α	β	γ
IC50 [nM]	12.20	4 523	9 583	45 442
SD [nM]	4.30	492	935	1438
Ratio to δ		371	785	3725
Kd [nM]	0.85	1 900	390	4 800

The selectivity of CPL302-253 was also tested in a broad panel of 468 human kinases at a single compound concentration (1 μM), using KINOMEScan technology. The high dose of the compound, which was more than 1000 times higher than its Kd for PI3Kδ was used in order to identify any interactions of CPL302-253 with human kinome. The obtained results showed that CPL302-253 has a unique selectivity towards PI3Kδ (Table 2). The only kinase to which the compound binds with affinity similar to its intended target is mutant PI3Kα isoform identified in cancer patients [20]. However, due to the low prevalence of the mutation the high affinity of the compound to the mutated kinase does not exclude usage of CPL302-253 as a selective PI3Kδ inhibitor. Similar to the results obtained earlier the binding affinity of CPL302-253 to PI3K isoforms α, β and γ was much lower than to isoform δ. There were no kinases other than members of PI3K which binding to specific substrate was inhibited by CPL302-253 more than 70%. 30% remaining binding is considered in KinomeScan assay as no off-target interaction.

**Table 2:** CPL302-253 interactions with human kinome. CPL302-253 binding to a human kinome was assessed in KINOMEScan assay at 1 μM. The numbers represent % of the remaining binding of specific ligand to kinase in competitive binding assay with CPL302-253. The table includes targets with remaining binding lower than 40% and PI3K isoforms.

Gene symbol	Remaining binding (%)
PIK3CA(H1047Y)	0
PIK3CD	1.1
PIK3CA(C420R)	12
ULK2	33
MEK5	35
ABL1-nonphosphorylated	36
PIK3CB	42
PIK3CA(I800L)	44
PIK3CA(E545A)	45
PIK3CA(E545K)	54
PIK3CA(E542K)	82
PIK3CA(H1047L)	92
PIK3CG	94
PIK3CA	96
PIK3CA(Q546K)	99
PIK3CA(M1043I)	100

**Fig 2:**
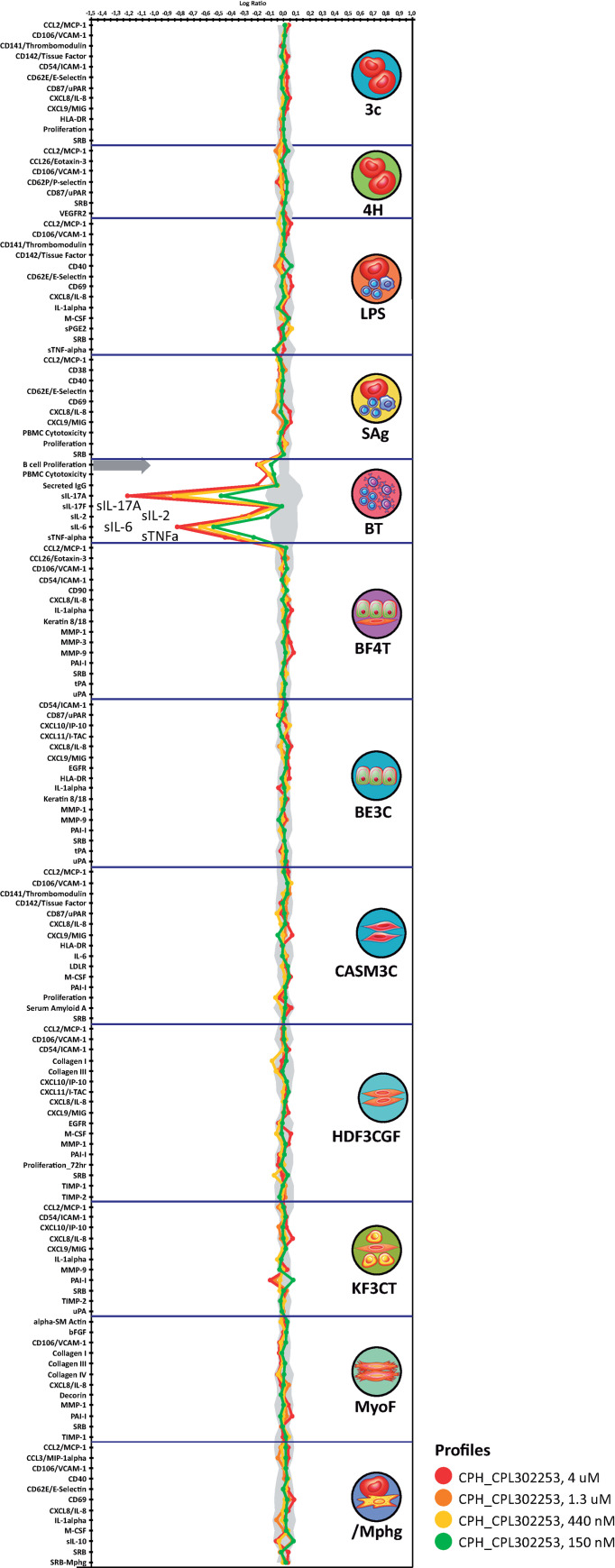
The influence of CPL302-253 on activated human primary cells functions. The influence of CPL302-253 on biomarker expression was assessed in the following human cell culture and co-culture systems: 3C system (HUVEC stimulated with IL-1β, TNFα and IFNγ), 4H system (HUVEC stimulated with IL-4 and histamine), LPS system (PBMC and HUVEC stimulated with LPS), SAg system (PBMC and HUVEC stimulated with TCR ligands), BT system (CD19 B cells and PBMC stimulated with αIgM and TCR ligands), BF4T system (bronchial epithelial cells and HDFn stimulated with TNFα and IL-4), BE3C system (bronchial epithelial cells stimulated with IL-1β, TNFα and IFNγ), CASM3C system (coronary artery smooth muscle cells stimulated with IL-1β, TNFα and IFNγ), HDF3CGF system (HDFn stimulated with IL-1, TNFα, IFNγ, EGF, bFGF and PDGF-BB), KF3CT system (keratinocytes and HDFn stimulated with IL-1β, TNFα, IFNγ and TGFβ), MyoF system (differentiated lung myofibroblasts stimulated with TNFα and TGFβ) and lymphsystem (HUVEC and M1 macrophages stimulated with Zymosan). Parameters measured are indicated along the x-axis. The results are shown as the log-transformed ratio of the biomarker readouts for the drug-treated sample to vehicle controls. The grey area parallel to the x-axis indicates the 95% significance region calculated based on historical vehicle controls. Biomarker activities are considered as changed when 2 or more consecutive concentrations change in the same direction relative to vehicle controls, are outside of the significance envelope, and have at least one concentration with an effect size > 20%. The anti-proliferative effect is indicated by a thick grey arrow.

Cultures of human primary cells allow studying an influence of potential drugs in conditions more relevant to *in vivo* experiments thancell lines. Of particular value are co-culture models in which interactions between cells can be studied. The BioMAP Diversity Plus platform consists of 12 culture and co-culture human primary cell system designed to model processes important for disease development. In particular BT system in which CD19 B cells and PBMC are stimulated with αIgM and TCR ligands allow to study processes important for autoimmune disease development. The IL-2, IL-6, IL-17 and TNFα, the biomarkers assessed in BT system, were shown to be influenced by PI3Kδ inhibitors [12]. The obtained results showed that CPL302-253 inhibits the expression of all of listed markers in BT system (Fig 2). In addition, the compound blocked proliferation of B cells what was also shown for other PI3Kδ inhibitors [12]. There was no impact of CPL302-253 on other studied systems, except the decrease of Plasminogen Activator Inhibitor-1 expression in co-culture of dermal fibroblasts and keratinocytes. However, the observed effect was lower than 0.2 log10 and induced only at concentrations as high as 150 nM.

CPL302-253 was tested in SafetyScan44 panel to study its influence on targets known to underline Adverse Drug Responses [21]. SafetyScan44 consists of assay designed to study the activity of several G Protein-Coupled Receptors (GPCR), transporters, ion channels, nuclear receptors, kinases and non-kinase enzymes which were shown to be responsible for off-target related side effects. The test was performed at 10 μM CPL302-253, the concentration 10 000 higher than Kd to intended therapeutic target. The results of each test in the panel are considered negative if the activation or inhibition of a given pathway does not exceed 70% of the control. The obtained results showed that CPL302-253 neither inhibits nor activates any of the studied pathways (Table 3). This data demonstrates that CPL302-253 has minor off-target potential, and shall be regarded as a safe compound in terms of biomarkers tested in SafetyScan44 panel.

**Table 3:** CPL302-253 selectivity in SafetyScan44 panel. Genetically modified reporter cell lines were treated with CPL302-253 at 10μM. G Protein-Coupled Receptors (GPCR), transporters, ion channels, nuclear receptors, kinases and non-kinase enzymes activity was measured in corresponding cell model. The CPL302-253 influence on measured parameter is indicated as % of response in control conditions.

Gene symbol	Assay mode	CPL302-253 10 μM % Response
**GPCRs**		
ADORA2A	Agonist	-0.8
ADORA2A	Antagonist	11.7
ADRA1A	Agonist	-22.5
ADRA1A	Antagonist	-2.8
ADRA2A	Agonist	0.4
ADRA2A	Antagonist	-0.7
ADRB1	Agonist	0.5
ADRB1	Antagonist	26.6
ADRB2	Agonist	0.5
ADRB2	Antagonist	17.7
AVPR1A	Agonist	-3.2
AVPR1A	Antagonist	-5.2
CCKAR	Agonist	-2.8
CCKAR	Antagonist	-2.4
CHRM1	Agonist	1.2
CHRM1	Antagonist	48.1
CHRM2	Agonist	8.2
CHRM2	Antagonist	35.2
CHRM3	Agonist	1.2
CHRM3	Antagonist	12.6
CNR1	Agonist	-9.2
CNR1	Antagonist	-1.3
CNR2	Agonist	16.4
CNR2	Antagonist	1.1
DRD1	Agonist	1.1
DRD1	Antagonist	9.3
DRD2S	Agonist	-4.5
DRD2S	Antagonist	-1.1
EDNRA	Agonist	-3.3
EDNRA	Antagonist	-4.8
HRH1	Agonist	-0.2
HRH1	Antagonist	7.2
HRH2	Agonist	0.6
HRH2	Antagonist	4.2
HTR1A	Agonist	5.6
HTR1A	Antagonist	-4.8
HTR1B	Agonist	8.0
HTR1B	Antagonist	0.1
HTR2A	Agonist	-1.5
HTR2A	Antagonist	11.5
HTR2B	Agonist	1.5
HTR2B	Antagonist	12.6
OPRD1	Agonist	-6.4
OPRD1	Antagonist	0.4
OPRK1	Agonist	-11.0
OPRK1	Antagonist	0.3
OPRM1	Agonist	-9.0
OPRM1	Antagonist	0.2
**Nuclear Hormone Receptors**		
AR	Agonist	-0.7
AR	Antagonist	8.5
GR	Agonist	0.0
GR	Antagonist	-5.6
**Transporters**		
DAT	Blocker	56.1
NET	Blocker	8.5
SERT	Blocker	21.5
**Ion Channels**		
CAV1.2	Blocker	-1.1
GABAA	Opener	-2.3
GABAA	Blocker	1.4
hERG	Blocker	-2.7
HTR3A	Opener	-0.8
HTR3A	Blocker	19.5
NAV1.5	Blocker	9.7
**Non-Kinase Enzymes**		
AChE	Inhibitor	27.8
COX1	Inhibitor	4.7
COX2	Inhibitor	11.9
MAOA	Inhibitor	8.5
PDE3A	Inhibitor	-8.1
PDE4D2	Inhibitor	2.0
**Kinases**		
INSR	Inhibitor	-6.5
LCK	Inhibitor	16.3
ROCK1	Inhibitor	10.3
VEGFR2	Inhibitor	30.7

CPL302-253 toxicity on lung epithelial cells was tested on Normal Human Bronchial Epithelial (NHBE) cells. The cells were incubated with different concentration of the compound. CPL302-253 effect on a broad panel of biomarkers was determined in order to cover diverse aspects of cell physiology. The lowest compound concentration affecting given parameter as well as AC50 value were determined. The obtained results showed (Table 4) that CPL302-253 does not influence any cell health parameter at concentration lower than 10 μM. The AC50 for all the tested parameters was higher than 100 μM.

Cell Health Parameter "# MEC (μM) AC50 (μM)

**Table 4:** CPL302-253 toxicity on NHB cells. The cells were cultured for 48 h in the presence of the compound. Cell health parameters were studied on fluorescent cell imager after staining. Minimum Effective Concentration (MEC) that alters the cell health parameters and AC50 (concentration at which 50% maximum effect is observed) in comparison to vehicle control was calculated for each cell health parameter.

Cell Health Parameter	Direction of Response	MEC [μM]	AC50 [μM]
Cell count	↓	50.6	>100
Nuclear size	↓	75.3	>100
DNA structure	↑	24.4	>100
Mitochondrial mass	↓	20.8	>100
Mitochondrial membrane potential	↑	22.8	>100
Oxidative stress	↑	16.1	>100
Glutathione content	↑	10.6	>100

**Fig 3:**
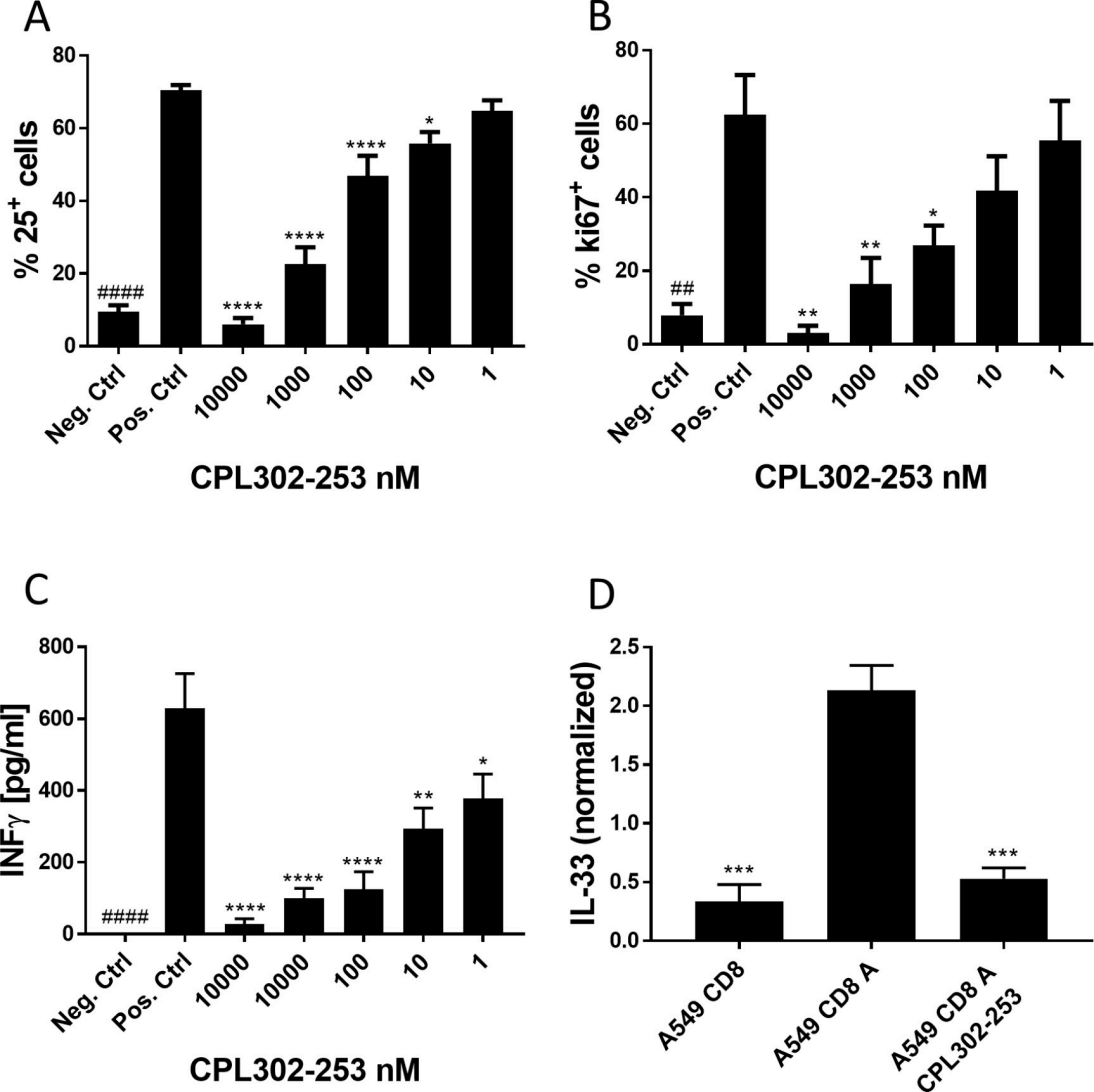
CLP302-253 blocks activation of primary lymphoid cells *in vitro*. (A and B) B cells were activated with ODN2006 and αIgM F(ab’)_2_ for 72 h. To some cultures, CPL302-253 was added. (A) Expression of and activation marker CD25 and (B) proliferation marker ki-67 was assessed by FACS. (C) CD8 T cells were activated with αCD2, αCD3 and αCD28. IFNγ release was measured after 48 h in culture supernatants by ELISA. (D) A549 cells were co-cultured with naïve CD8 or activated CD8 cells. To some cultures CPL302-253 was added at 1 μM. IL-33 expression was assessed after 72 h of treatment in CD8 and A549 cell lysates by ELISA. For each experiment at least three independent biological replicates were performed. p-value < 0.05 was regarded as significant response. * p-value < 0.05; ** p-value <0.01; *** p-value <0.001. Neg. Ctrl–naïve cells, Pos. Ctrl–activated cells.

Since PI3Kδ regulates lymphocyte activation, we tested whether CPL302-253 is able to stop this process. CD25 is a well-established marker of lymphocyte activation. CPL302-253 blocked CD25 expression by B cell after BCR and Toll-Like Receptor (TLR) stimulation. We observed inhibition of CD25 expression at a concentration as low as 10 nM (Fig 3A). The compound blocked also B cell proliferation (Fig 3B) what was measured by means of ki-67 expression. We measured also how CPL302-253 influences IFNγ expression by CD8 T cells after T Cell Receptor (TCR) engagement. The production of the cytokine was blocked by the compound at concentration as low as 1 nM (Fig 3C). The obtained results show that CPL302-253 efficiently blocks lymphocyte functions *in vitro*. Since IL-33 is an important asthma mediator we tested whether CPL302-253 blocks this cytokine production in epithelial and T cell co-cultures, where CD8 lymphocytes induce IL-33 expression by A549 cells. Indeed, we observed strong inhibition of IL-33 expression in co-cultures treated with the compound (Fig 3D).

**Fig 4:**
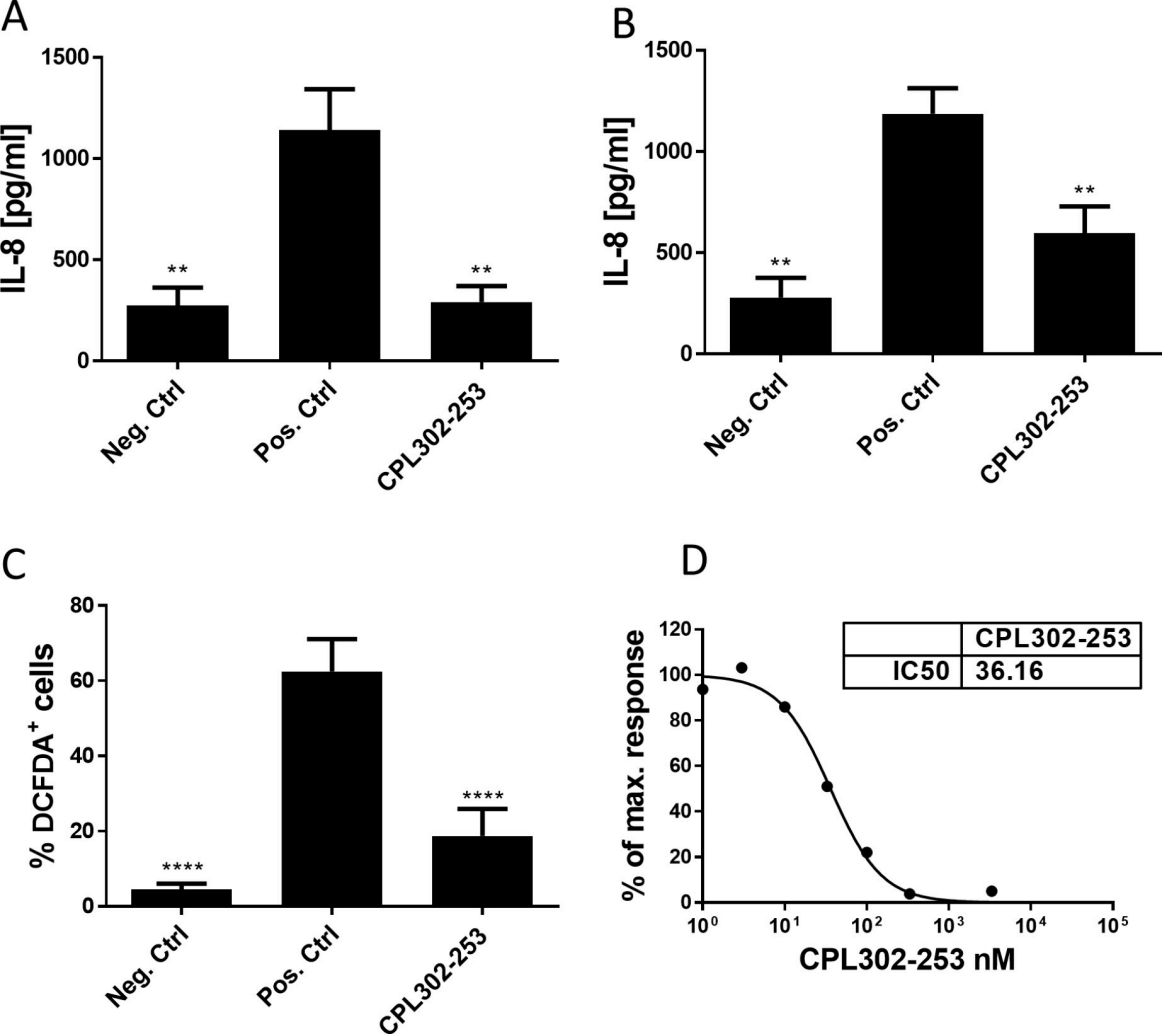
CPL302-253 blocks activation of primary myeloid cells *in vitro*. (A, B) Human primary neutrophils were activated with (A) 100 μg/ml CSE or (B) 1 μg/ml LPS. After 6 h of incubation culture supernatants were collected and IL-8 release was assessed by ELISA. (C) ROS accumulation was analysed after 2 h GM-CSF (20ng/ml) and LPS (1 μg/ml) stimulation. % of neutrophils stained positively by ROS fluorescent indicator were determined by FACS. (D) Human whole blood was stimulated with antibody specific for the high-affinity IgE receptor FcεRI. After 25 min of incubation basophils were stained and identified on FACS based on light scatter and CXCR3 expression. Basophil activation was assessed based on CD63 activation marker expression. For each experiment at least three independent biological replicates were performed. p-value < 0.05 was regarded as significant response. * p-value < 0.05; ** p-value <0.01; *** p-value <0.001; **** p-value <0.001. Neg. Ctrl–naïve cells, Pos. Ctrl–activated cells.

CSE stimulated neutrophils produce IL-8 (Fig 4A) or LPS (Fig 4B). A significant increase inIL-8 production was observed after 6 h CSE or LPS exposure. CPL302-253 reduced IL-8 production after both stimulation protocols. Reactive Oxygen Species (ROS) are an important element of anti-bacterial immune response, however, dysregulated ROS production plays a role in lung function worsening in asthmatic patients. Neutrophils are the major source of ROS, therefore the influence of CPL302-253 on ROS production was assessed. CPL302-253 reduced ROS accumulation in neutrophils after GMCSF/LPS stimulation (Fig 4C). Upon cross-linking of high-affinity IgE receptor FcεRI, basophils are activated and express CD63 activation marker. The effect of CPL302-253 was measured in a dose range of the compound. CPL302-253 blocks CD63 expression after basophils activation with IC50 36 nM (Fig 4D).

**Fig 5:**
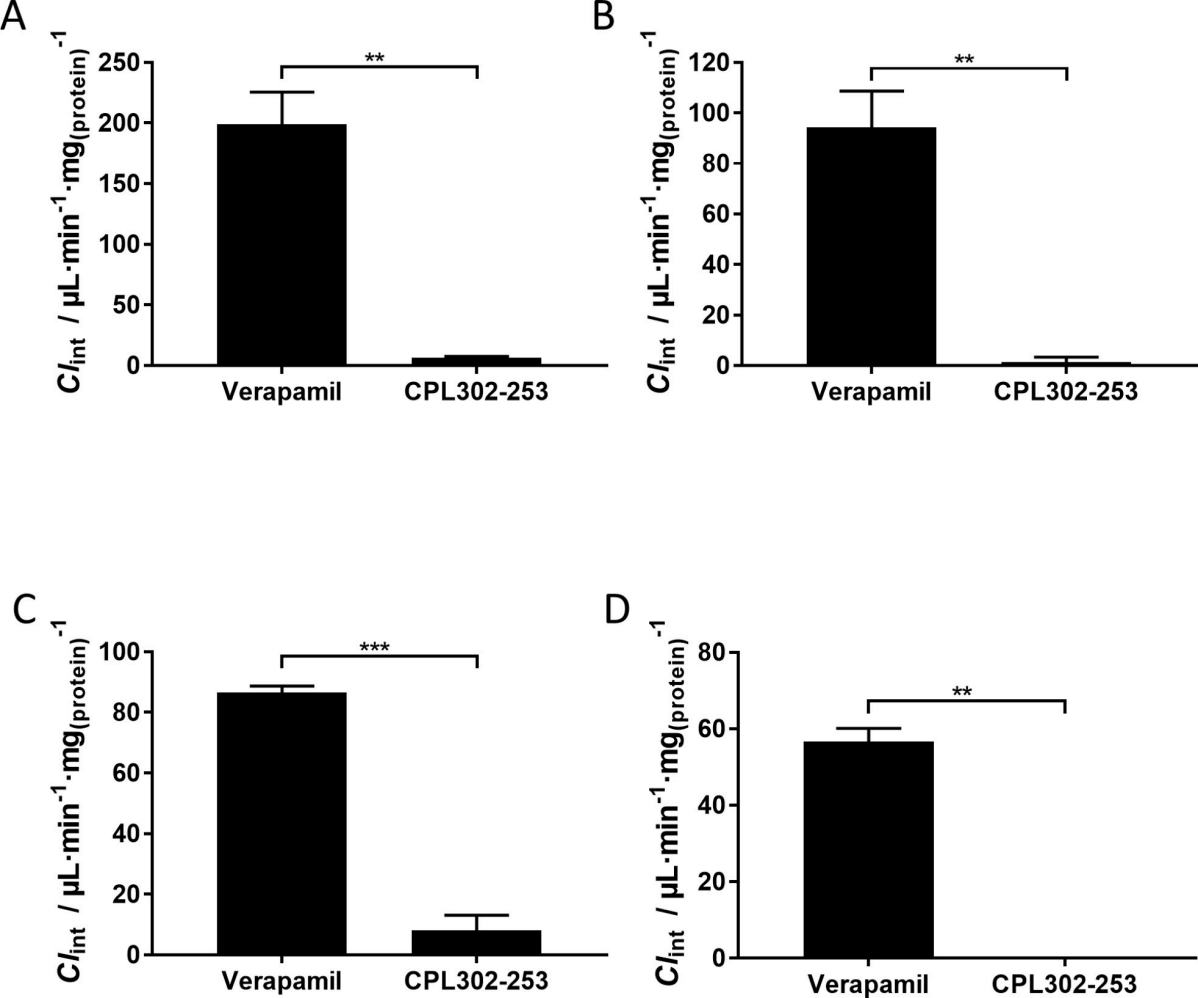
CPL302-253 is metabolically stable. CPL302-253 metabolic stability was assessed in mouse microsomes (A), human microsomes (B), mouse cryopreserved hepatocytes (C) and human cryopreserved hepatocytes (D), *Cl*_int_ was calculated using the rate of compound disappearance from the reaction.

Metabolic stability *in vitro* is one of the measures used to predict *in vivo* PK. Since metabolic stability *in vitro* (expressed as *Cl*_int_) is related to metabolic clearance *in vivo*, selection of stable compounds at early stage of drug development increases the chance for favourable PK properties [22]. CPL302-253 stability was assessed in mouse and human microsomes and cryopreserved hepatocytes. In both studies verapamil was used as a reference compound with high degradation rate [23, 24]. *Cl*_int_ of CPL302-253 in microsomes was nearly 30 or 70 times lower when compared to verapamil in mouse and human microsomes (Fig 5A and 5B), thus indicating that the compound is unlikely to undergo extensive phase I transformations. Similarly, CPL302-253 degradation in mouse hepatocytes was 11-fold slower as compared to verapamil, whereas in human hepatocytes degradation was not observed at all during the time course of the experiment (80 min) (Fig 5C and 5D). Primary hepatocytes contain much broader spectrum of metabolic enzymes than microsomes, including phase I, phase II and phase III enzymes, and are a reliable model for extrapolation of degradation rate to *in vivo* conditions. The presented results show that CPL302-253 is metabolically stable in both microsomes and hepatocytes, and thus make this molecule a promising drug candidate with low expected hepatic clearance *in vivo*.

**Fig 6:**
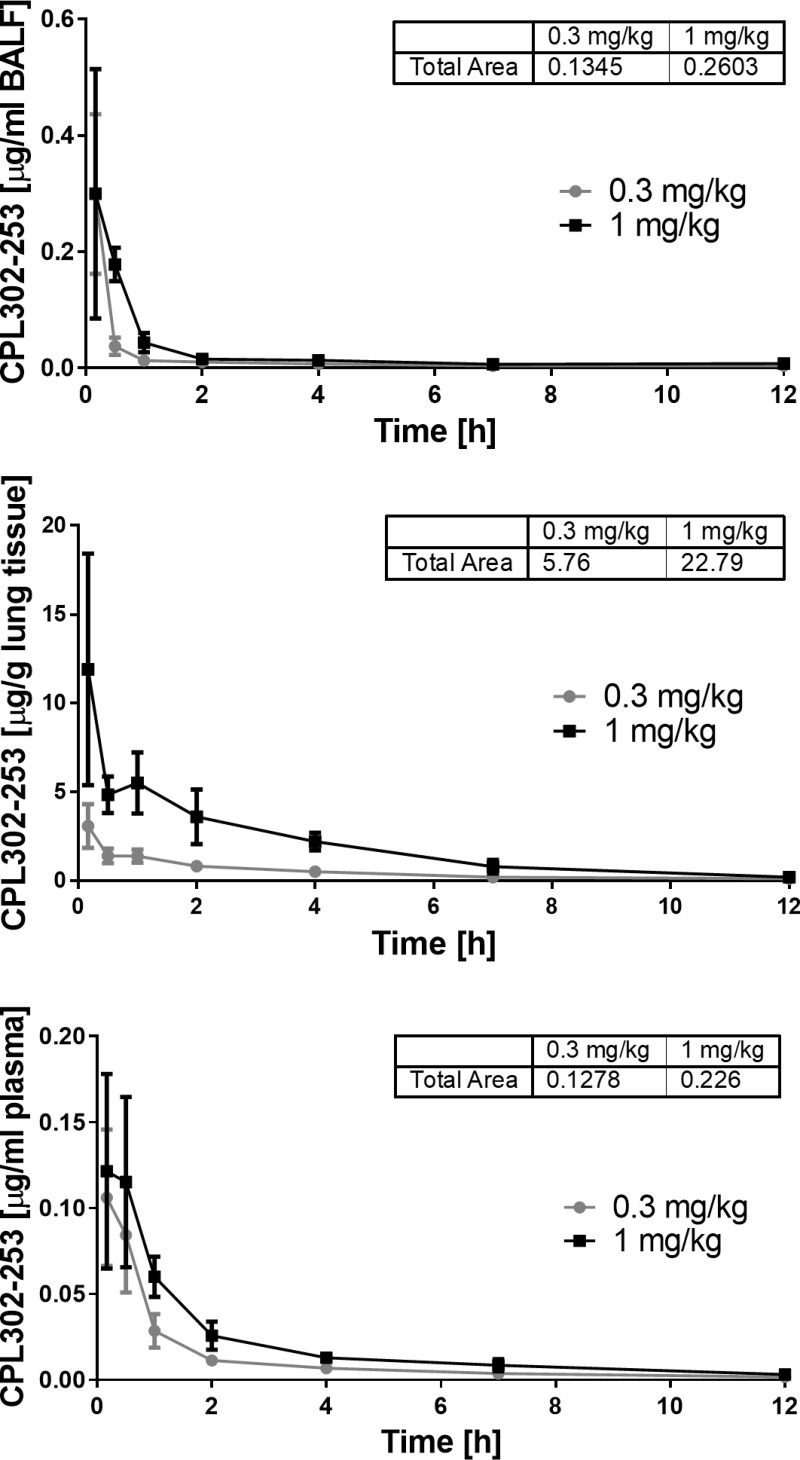
CPL302-253 accumulates in lungs after intratracheal administration. The compound concentration was determined in BALF (A), lungs (B) and plasma (C) of Balb/c mice at different time-points after intratracheal administration.

Direct lung administration of therapeutic agents in inflammatory lung disease allows to decrease systemic side effects related to inhibition of immune response. In order to determine CPL302-253 exposure upon the lung delivery we performed PK studies in mice using intratracheal route of administration, which mimics the inhalation in humans [25]. The compound concentration was measured at different time-points during 12-hour sampling period. The obtained results showed that CPL302-253 is exclusively accumulated in the lung tissue and BAL with minor penetration to the circulation (Fig 6). This data shows that inhibition of PI3Kδ signalling by CPL302-253 can occur only in lungs since the compound is moderately present in other compartments and makes it a solid candidate for therapeutic inhalation administration.

**Fig 7:**
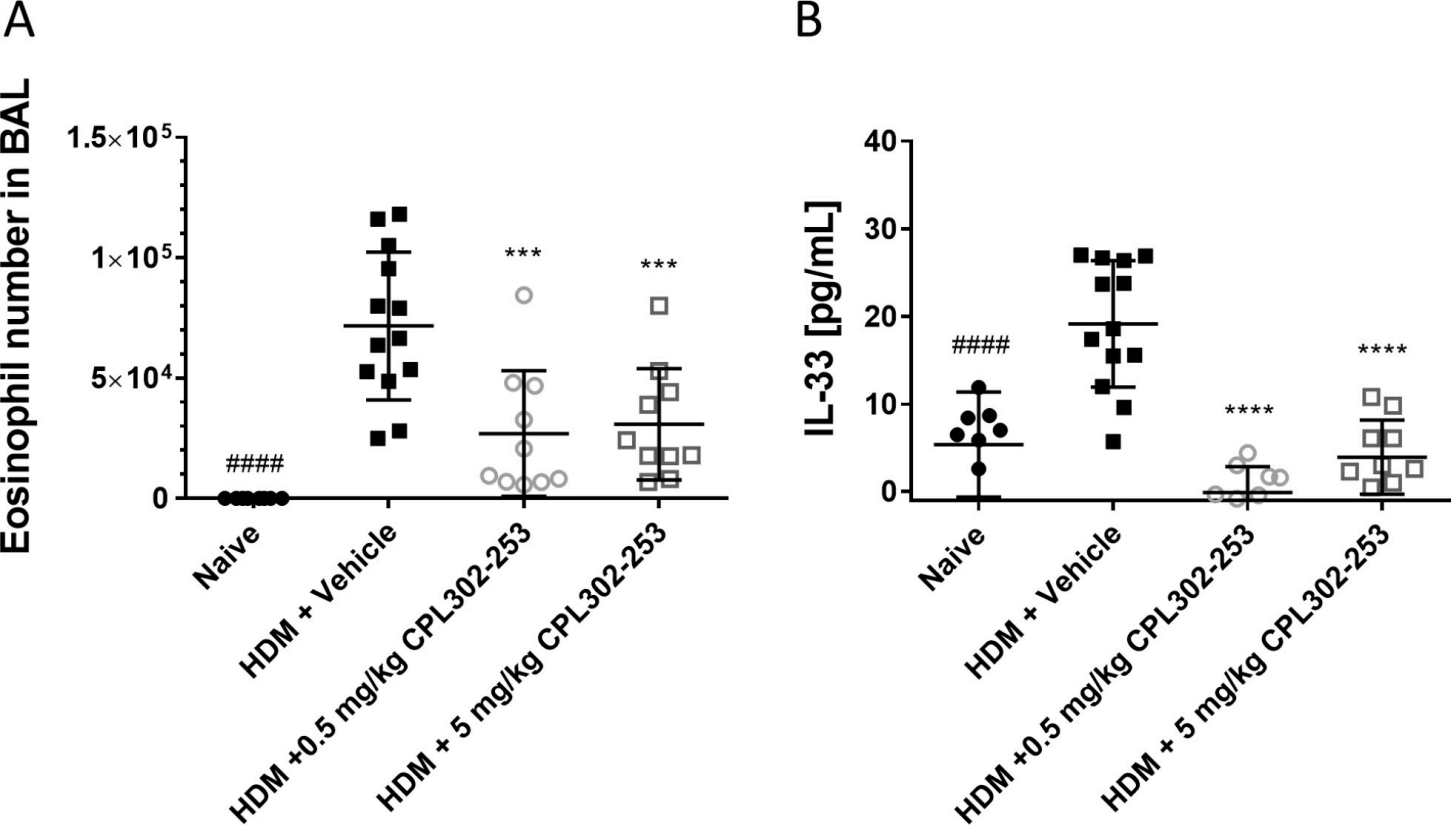
CPL302-253 is bocks eosinophil infiltration to the lungs and IL-33 expression in HDM induced asthma mouse model. Balb/c mice were immunized by s.c. route with CFA mixed with HDM lyophilized extract or saline. On day 12, 13 and 14 the mice were administrated by i.n. route with CPL302-253 or saline. On day 14, 60 min after compound administration the animals were challenged by i.n. route with HDM in sterile saline or saline. On day 15 eosinophils number (A) and IL-33 concentration (B) were measured in BAL.

The effectiveness of CPL302-253 was studied in HDM-induced severe asthma mouse model in which animals were sensitized with single s.c. injection of HDM mixed with CFA. After 12 days upon sensitization, animals were treated for 3 consecutive days with CPL302-253 by i.n. route and subsequently challenged with HDM by i.n. route. The accumulation of eosinophils and IL-33 concentration were analysed in BALF. The obtained results showed that HDM challenge in the described model led to a profound influx of eosinophils to the lungs as well as significant up-regulation of IL-33 in the BALF (Fig 7). This proves that the employed experimental procedure is suitable for induction of immune response characteristic for asthma. Upon administration of CPL302-253 it has effectively blocked eosinophil influx as well as IL-33 production in the lungs of treated animals. The obtained results show that the compound is not only effective *in vivo* but also stops asthma specific immune response in the validated disease model.

## Discussion

Although allergic asthma in the majority of cases can be controlled with available therapies, still there is significant number (e.g. corticosteroid insensitive or corticosteroid resistant) of patients for which there is no effective therapeutic option available. PI3Kδ is considered as a potential new therapeutic target for difficult to treat asthma [26]. This kinase participates in the regulation of multiple inflammatory processes, many of which have been shown to play direct role in asthma development. The broad and strong effect of PI3Kδ on immune response makes it especially attractive target for severe asthma treatment for which there is no effective cure available. Since PI3Kδ is expressed almost exclusively in immune cells, inhibition of this kinase should have limited effect on other cell types. On the other hand, the available data regarding side effects of Idelalisib, the PI3Kδ inhibitor which is used in leukaemia treatment, show that systemic inhibition of this kinase is associated with the strong toxicity.

It is postulated that reasons behind severe side effects of Idelalisib could be related to limited selectivity of the compound and/or systemic administration [9]. Therefore, better selectivity or site-specific administration should be considered as important characteristics of new PI3Kδ inhibitors, which are under development for inflammatory disease treatment. Based on the generated results, limited systemic exposure of CPL302-253 after pulmonary administration may be beneficial for its safety upon inhalation administration.

In this work, we present CPL302-253, a highly selective PI3Kδ inhibitor. CPL302-253 had a fold selectivity to PI3Kδ over other PI3K isoforms measured by IC50 in kinase assay (Table 1). Furthermore it presents much higher than fold selectivity of Idelalisib, duvelisib and copanlisib [27]. Kd determination in KinomeScan assay further proved that CPL302-253 is highly selective towards PI3Kδ. KinomeScan studies in broad panel of 468 human kinases showed no off-target interactions of CPL302-253 at 1 μM concentration, which is considered a good prognostic regarding the compound safety.

To study the influence of substances on a broad range of human immune and non-immune primary cell cultures and co-cultures, BioMap platform was used. CPL302-253 interfered with maker molecule expression or physiological functions almost exclusively in co-culture model of immune cells, in particular T cells and B cells activated with BCR and TCR activators, what is consistent with other PI3Kδ inhibitors profiles in this study. In addition, CPL302-253 showed limited influence on culture and co-culture systems consisting non-immune cells in comparison to other PI3Kδ inhibitors further demonstrating its high isotype selectivity [12]. BioMap provided also first prove of CPL302-253 effectiveness in *in vitro* models. Among markers of B cells and T cells activation measured in BT co-culture IL-2, IL-6, IL-17 and TNFα expression were significantly blocked by CPL302-253. The molecule inhibited B cell proliferation, further proving its anti-inflammatory potential. There were no cytotoxic effects observed in any BioMap culture systems which is considered a good prognostic regarding the compound toxicity.

CPL302-253 was examined in SafetyScan44, a panel of GPCR, transporters, nuclear receptors, kinases and non-kinase enzymes, which were shown to be important mediators of drug-induced toxicity [21]. The obtained results showed that the compound neither inhibits nor activates any of the studied pathways. The human Ether-àgo-go-Related Gene (hERG) channel, one of the molecules which activity is tested in SafetyScan44, is important controller of electrical activity of the heart. Available preclinical data regarding Nemiralisib, PI3Kδ inhibitor recently withdrawn from developmental program, showed that this compound has activity towards hERG [28]. Therefore, the lack of influence on hERG is especially good prognostics for further development of CPL302-253 as the drug candidate.

CPL302-253 blocked immune functions of different immune-cells tested. Activated B cells stoped to express CD25 activation marker if treated with CPL302-253. The role of PI3Kδ in the regulation of lymphocyte proliferation is well established and CPL302-253 blocked it in activated B cells. Cytokines production is one of the important hallmarks of T cells activation and CPL302-253 blocked IFNγ production by CD8 T cells. In co-culture model of lung epithelial cell line A549 and activated CD8 T cells CPL302-253 blocked IL-33 expression. Since CD8 T cells are necessary for induction of IL-33 in this model and PI3Kδ regulates CD8 T cell function, it is reasonable to consider, that inhibition of IL-33 expression in this model occurs due to CD8 T cell activity blockade. However, taking under account the reports regarding role of PI3Kδ in cytokine expression by lung epithelial cells [5] such a statement requires further evidence.

PI3Kδ regulates also functions of immune cells of myeloid lineage and CPL302-253 is effective in these cells as well. CSE- or LPS-activated neutrophils block to produce IL-8, the known lung inflammation mediator if treaded with CPL302-253 *in vitro*. We have proved that compound can block ROS production by neutrophils. Basophils, another immune cell population mediating inflammatory lung disease, express CD63 marker when activated. In our studies CPL302-253 efficiently blocked CD63 expression by activated basophils. Taken together, the results show that CPL302-253 is a potent modulator of various immune cells.

Site-specific administration is commonly used to restrict systemic side effects of therapeutics. In order to induce therapeutic effect, the drugs need to reach the target organ after site-specific administration. In order to limit distribution through the body the substances need to retain in the site of administration. Therefore, we tested body distribution of CPL302-253 after intratracheal administration in mouse model. The obtained results showed that the compound accumulates in the lungs of the animals and maintains relevant concentration several hours after administration. The substance has limited systemic availability after intratracheal administration since its concentration is two orders of magnitude lower in the blood than in the lungs at any time-point after administration. This data shows that CPL302-253 has a potential of limited toxicity due to site-specific restriction of its actions.

OVA-induced (OVA model) allergic asthma is the most common model used to study drug efficacy in the preclinical setting. However, this model is associated with several limitations caused by simple chemical structure of the antigen. This model was developed in the time when our understanding of asthma development was much more limited and the role of innate cells in this process was not yet recognized. The more complex antigens, containing PAMP’s and enzymes leading to epithelial layer disruption outrank OVA model by induction of both, adaptive and innate mechanisms of asthma development. This was also observed in HDM asthma models [29]. In the light of data the translation of the data generated using OVA model has been questioned [17, 30]. For the purpose of this study new HDM sensitization protocols was developed which allows time and cost-effective testing of potential asthma drugs. The animals in this model receive by parenteral route single sensitization with HDM dispensed in Complete Freund’s adjuvant. After 2 weeks single i.n. administration of HDM leads to profound accumulation of eosinophils in the lungs as well as IL-33 expression upregulation. The limited number of i.n. allergen administrations make this model especially suitable for tests of compounds administrated directly to the lungs. This model allowed us to test CPL302-253 efficacy *in vivo* after i.n. administration. We showed that our compound is effective, safe and effectively blocks the important for asthma development inflammatory processes like lung eosinophils accumulation and up-regulation of IL-33 expression. Eosinophils play important role in asthma exacerbation [31–33]. It was shown recently that IL-33 is an important asthma exacerbation mediator [34–36]. Therefore, it is likely that CPL302-253 will be effective in treatment and prevention of asthma.

Concluding, we presented *in vitro* and *in vivo* results of a novel, potent and selective PI3Kδ inhibitor, CPL302-253, supporting the rationale of its development in immune-related lung diseases as an inhaled agent. Currently the compound undergoes further evaluation as the drug candidate.

## Supporting information

S1 Data (XLSX)

## References

[1] Barnes PJ (2008) Immunology of asthma and chronic obstructive pulmonary disease. Nat Rev Immunol 8: 183–192. 10.1038/nri2254 18274560

[2] Lambrecht BN, Hammad H (2015) The immunology of asthma. Nat Immunol 16: 45–56. 10.1038/ni.3049 25521684

[3] Morita H, Moro K, Koyasu S (2016) Innate lymphoid cells in allergic and nonallergic inflammation. J Allergy Clin Immunol 138: 1253–1264. 10.1016/j.jaci.2016.09.011 27817797

[4] Okkenhaug K, Vanhaesebroeck B (2003) PI3K in lymphocyte development, differentiation and activation. Nat Rev Immunol 3: 317–330. 10.1038/nri1056 12669022

[5] Jeong JS, Kim JS, Kim SR, Lee YC (2019) Defining Bronchial Asthma with Phosphoinositide 3-Kinase Delta Activation: Towards Endotype-Driven Management. Int J Mol Sci 20. 10.3390/ijms20143525 PMC6679152 31323822

[6] Zirlik K, Veelken H (2018) Idelalisib. Recent Results Cancer Res 212: 243–264. 10.1007/978-3-319-91439-8_12 30069634

[7] Blair HA (2018) Duvelisib: First Global Approval. Drugs 78: 1847–1853. 10.1007/s40265-018-1013-4 30430368

[8] Okabe S, Tanaka Y, Tauchi T, Ohyashiki K (2019) Copanlisib, a novel phosphoinositide 3-kinase inhibitor, combined with carfilzomib inhibits multiple myeloma cell proliferation. Ann Hematol 98: 723–733. 10.1007/s00277-018-3547-7 30430191

[9] Greenwell IB, Ip A, Cohen JB (2017) PI3K Inhibitors: Understanding Toxicity Mechanisms and Management. Oncology (Williston Park) 31: 821–828. 29179250

[10] Barnes PJ (2010) New therapies for asthma: is there any progress? Trends Pharmacol Sci 31: 335–343. 10.1016/j.tips.2010.04.009 20554041

[11] Hoegenauer K, Soldermann N, Zecri F, Strang RS, Graveleau N, et al (2017) Discovery of CDZ173 (Leniolisib), Representing a Structurally Novel Class of PI3K Delta-Selective Inhibitors. ACS Med Chem Lett 8: 975–980. 10.1021/acsmedchemlett.7b00293 28947947 PMC5601375

[12] Allen RA, Brookings DC, Powell MJ, Delgado J, Shuttleworth LK, et al (2017) Seletalisib: Characterization of a Novel, Potent, and Selective Inhibitor of PI3Kdelta. J Pharmacol Exp Ther 361: 429–440. 10.1124/jpet.116.237347 28442583

[13] Rao VK, Webster S, Dalm V, Sediva A, van Hagen PM, et al (2017) Effective "activated PI3Kdelta syndrome"-targeted therapy with the PI3Kdelta inhibitor leniolisib. Blood 130: 2307–2316. 10.1182/blood-2017-08-801191 28972011 PMC5701526

[14] Wilson R, Jarvis E, Montembault M, Hamblin JN, Hessel EM, et al (2018) Safety, Tolerability, and Pharmacokinetics of Single and Repeat Doses of Nemiralisib Administered via the Ellipta Dry Powder Inhaler to Healthy Subjects. Clin Ther 40: 1410–1417. 10.1016/j.clinthera.2018.06.011 30055824

[15] Nelson RP Jr., DiNicolo R, Fernandez-Caldas E, Seleznick MJ, Lockey RF, et al (1996) Allergen-specific IgE levels and mite allergen exposure in children with acute asthma first seen in an emergency department and in nonasthmatic control subjects. J Allergy Clin Immunol 98: 258–263. 10.1016/s0091-6749(96)70148-3 8757201

[16] Woo LN, Guo WY, Wang X, Young A, Salehi S, et al (2018) A 4-Week Model of House Dust Mite (HDM) Induced Allergic Airways Inflammation with Airway Remodeling. Sci Rep 8: 6925 10.1038/s41598-018-24574-x 29720689 PMC5932037

[17] Aun MV, Bonamichi-Santos R, Arantes-Costa FM, Kalil J, Giavina-Bianchi P (2017) Animal models of asthma: utility and limitations. J Asthma Allergy 10: 293–301. 10.2147/JAA.S121092 29158683 PMC5683778

[18] Drewry DH, Wells CI, Andrews DM, Angell R, Al-Ali H, et al (2017) Progress towards a public chemogenomic set for protein kinases and a call for contributions. PLoS One 12: e0181585 10.1371/journal.pone.0181585 28767711 PMC5540273

[19] Berg EL, Kunkel EJ, Hytopoulos E, Plavec I (2006) Characterization of compound mechanisms and secondary activities by BioMAP analysis. J Pharmacol Toxicol Methods 53: 67–74. 10.1016/j.vascn.2005.06.003 16040258

[20] Cohen Y, Goldenberg-Cohen N, Shalmon B, Shani T, Oren S, et al (2011) Mutational analysis of PTEN/PIK3CA/AKT pathway in oral squamous cell carcinoma. Oral Oncol 47: 946–950. 10.1016/j.oraloncology.2011.07.013 21824802

[21] Bowes J, Brown AJ, Hamon J, Jarolimek W, Sridhar A, et al (2012) Reducing safety-related drug attrition: the use of in vitro pharmacological profiling. Nat Rev Drug Discov 11: 909–922. 10.1038/nrd3845 23197038

[22] Masimirembwa CM, Bredberg U, Andersson TB (2003) Metabolic stability for drug discovery and development: pharmacokinetic and biochemical challenges. Clin Pharmacokinet 42: 515–528. 10.2165/00003088-200342060-00002 12793837

[23] Obach RS (1999) Prediction of human clearance of twenty-nine drugs from hepatic microsomal intrinsic clearance data: An examination of in vitro half-life approach and nonspecific binding to microsomes. Drug Metab Dispos 27: 1350–1359. 10534321

[24] Williamson B, Wilson C, Dagnell G, Riley RJ (2017) Harmonised high throughput microsomal stability assay. J Pharmacol Toxicol Methods 84: 31–36. 10.1016/j.vascn.2016.10.006 27773845

[25] Turner PV, Brabb T, Pekow C, Vasbinder MA (2011) Administration of substances to laboratory animals: routes of administration and factors to consider. J Am Assoc Lab Anim Sci 50: 600–613. 22330705 PMC3189662

[26] Yoo EJ, Ojiaku CA, Sunder K, Panettieri RA Jr. (2017) Phosphoinositide 3-Kinase in Asthma: Novel Roles and Therapeutic Approaches. Am J Respir Cell Mol Biol 56: 700–707. 10.1165/rcmb.2016-0308TR 27977296 PMC5516292

[27] Lampson BL, Brown JR (2017) PI3Kdelta-selective and PI3Kalpha/delta-combinatorial inhibitors in clinical development for B-cell non-Hodgkin lymphoma. Expert Opin Investig Drugs 26: 1267–1279. 10.1080/13543784.2017.1384815 28945111 PMC5747968

[28] Down K, Amour A, Baldwin IR, Cooper AW, Deakin AM, et al (2015) Optimization of Novel Indazoles as Highly Potent and Selective Inhibitors of Phosphoinositide 3-Kinase delta for the Treatment of Respiratory Disease. J Med Chem 58: 7381–7399. 10.1021/acs.jmedchem.5b00767 26301626

[29] Gregory LG, Lloyd CM (2011) Orchestrating house dust mite-associated allergy in the lung. Trends Immunol 32: 402–411. 10.1016/j.it.2011.06.006 21783420 PMC3381841

[30] Chapman DG, Tully JE, Nolin JD, Janssen-Heininger YM, Irvin CG (2014) Animal models of allergic airways disease: where are we and where to next? J Cell Biochem 115: 2055–2064. 10.1002/jcb.24881 25043224 PMC4199895

[31] Price DB, Rigazio A, Campbell JD, Bleecker ER, Corrigan CJ, et al (2015) Blood eosinophil count and prospective annual asthma disease burden: a UK cohort study. Lancet Respir Med 3: 849–858. 10.1016/S2213-2600(15)00367-7 26493938

[32] Denlinger LC, Phillips BR, Ramratnam S, Ross K, Bhakta NR, et al (2017) Inflammatory and Comorbid Features of Patients with Severe Asthma and Frequent Exacerbations. Am J Respir Crit Care Med 195: 302–313. 10.1164/rccm.201602-0419OC 27556234 PMC5328178

[33] Green RH, Brightling CE, McKenna S, Hargadon B, Parker D, et al (2002) Asthma exacerbations and sputum eosinophil counts: a randomised controlled trial. Lancet 360: 1715–1721. 10.1016/S0140-6736(02)11679-5 12480423

[34] Snelgrove RJ, Gregory LG, Peiro T, Akthar S, Campbell GA, et al (2014) Alternaria-derived serine protease activity drives IL-33-mediated asthma exacerbations. J Allergy Clin Immunol 134: 583–592 e586. 10.1016/j.jaci.2014.02.002 24636086 PMC4152000

[35] Jackson DJ, Makrinioti H, Rana BM, Shamji BW, Trujillo-Torralbo MB, et al (2014) IL-33-dependent type 2 inflammation during rhinovirus-induced asthma exacerbations in vivo. Am J Respir Crit Care Med 190: 1373–1382. 10.1164/rccm.201406-1039OC 25350863 PMC4299647

[36] Castanhinha S, Sherburn R, Walker S, Gupta A, Bossley CJ, et al (2015) Pediatric severe asthma with fungal sensitization is mediated by steroid-resistant IL-33. J Allergy Clin Immunol 136: 312–322 e317. 10.1016/j.jaci.2015.01.016 25746970 PMC4534777

